# An assessment of the implications of distribution remuneration and taxation policies on the final prices of prescription medicines: evidence from 35 countries

**DOI:** 10.1007/s10198-024-01706-x

**Published:** 2024-09-19

**Authors:** Giovanny Leon, Christophe Carbonel, Aparajit Rampuria, Ravindra Singh Rajpoot, Parth Joshi, Panos Kanavos

**Affiliations:** 1 Price Governance and Negotiations, Value and Access and Commercial Development, Novartis Pharma AG, Basel, Switzerland; 2 Value and Access, Novartis Healthcare Private Limited, Hyderabad, India; 3 Department of Health Policy and Medical Technology Research Group-LSE Health, The London School of Economics and Political Science, Houghton Street, London, WC2A 2AE UK

**Keywords:** National drug policy, Drug distribution, Drug taxation, Regressive mark ups, Regulation, Supply systems, Impact assessment

## Abstract

This paper analyses the structure of and variability in taxation and prescription drug distribution policies and quantifies the impact of such policies on the cost of prescription drugs to health systems in 35 countries. Taxes on prescription drugs remain highly prevalent (83% of the sample) although 63% of the sample countries implement a lower than standard VAT rate. Three remuneration types of the wholesale and retail distribution chain have been identified. Wholesale and retail distributors are remunerated on a regressive mark-up basis, which is price-dependent, although fixed fees and fixed percentages, which are non-price dependent, are also highly prevalent. Price component analysis for three groups of products classed as high-, medium- and low-priced suggests that mark-ups plus taxes varied significantly across countries and products, and ranged from 5% to 187% of ex-factory prices. Average margins also vary significantly by countries and products ranging 5–65% of retail prices. The cost of distribution and taxation contributes significantly to prescription drug costs for health systems. Although distribution chain remuneration raises efficiency and overall affordability questions, these need to be considered together with the regulatory framework shaping market structure of the distribution chain, as well as any prevailing horizontal and vertical integration policies. The overall cost of prescription drugs could be reduced immediately by eliminating taxation; this could go some way to alleviate fiscal pressures on health budgets, whilst avoiding resource re-allocation from health to other sectors.

**Supplementary Information:**

The online version contains supplementary material available at 10.1007/s10198-024-01706-x.

## Background

The global expenditure on prescription drugs alone is expected to reach US$1.6 trillion by 2025 [1]. That cost contributes to rising overall healthcare expenditure and has been a major concern among the healthcare decision-makers at global level [2, 3]. A variety of cost control measures have been introduced in most developed, transition or middle income countries to curb the rate of growth in drug spend aiming to meet macroeconomic efficiency targets as well as improve affordability and access to prescription drugs [4–8].

Within that context, a stable and efficient supply system whereby prescription drugs reach patients in a timely fashion, has been one of the key priority areas of national drug policies [9]. The World Health Organisation (WHO) has commented on supply systems being key contributors to access and rational drug use [10].

Regulation of remuneration mark-ups across the pharmaceutical distribution chain and taxes levied by governments on prescription drugs are two such policies that contribute significantly to retail drug price levels paid for by health insurers and/or patients. The 2020 WHO guidelines on country pharmaceutical pricing policies included regulation of mark-ups across the pharmaceutical supply and distribution chain, with an aim to reduce the variability in prescription drug prices through clear pricing rules [11, 12]. Despite that, there is limited evidence on the structure of the distribution chain, the nature and size of distribution mark-ups, both at wholesale and pharmacy level, taxation regulations, and their impact on the final (retail) drug prices paid for by health care systems [7, 8, 13–18]. Much of the existing evidence focuses on describing the distribution mark-up structure in the European region where most countries regulate wholesaling and retailing [7, 8, 13, 14] and is largely outdated [16]. Additionally, few studies focus on specific settings, including low-income countries, or evaluate the impact on prices for generic medications [15, 18, 19]. Despite recent efforts to systematically capture wholesale and pharmacy mark-ups [7, 20], the financial impact that distribution and taxation policies place on healthcare systems, has not been quantified adequately [7, 17, 20, 21].

In light of the above, the objective of this paper is twofold: first, to analyse the structure and variability in prescription drug distribution and taxation policies across selected settings; and second, to quantify the impact of such policies on the cost of prescription drugs to health systems and/or patients in these settings. In addressing these objectives, the paper makes a distinct contribution to the literature in two ways: first, it provides a comparative synthesis and analysis of the approaches used in countries that regulate distribution (wholesale and pharmacy) mark ups and implement taxes on the consumption of prescription drugs; and second, it assesses the impact these policies have on the cost of prescription drugs to health care systems and patients. Our study does not address the level of mark ups across settings, and whether these are optimal or not; answering this question would require an investigation into geographical and/or population criteria regarding the location of, particularly, retail outlets, or the extent to which horizontal or vertical integration policies are implemented in different settings, all of which are beyond the scope of the current study.

## Methods

### Scope

We focused on intervention policies and practices relating to the wholesale and retail distribution, and taxation rates of retail prescription drugs in select settings. The focus on the retail market is justified by its size as a proportion of the total pharmaceutical market. The term “wholesale and retail distribution policies and practices” included mark-ups (regressive percentages, regressive fees, or a combination of regressive percentages with fees), fixed remuneration (which could take the form of flat fees or fixed percentages [the latter known as linear mark-ups]), and dispensing fees or charges. Box 1 provides a list of terms and their associated definitions.

Box 1: Glossary of terms used

**Table:** 

Term	Definition
Ex-factory Price	• The manufacturer's posted price of a pharmaceutical or other products. Also referred to as manufacturer price, ex-manufacturer price, or manufacturer's selling price
Retail pharmacy price	• The price charged by community pharmacies to the general public, including any pharmacy remuneration such as a pharmacy mark-up or dispensing fee
Retailer	• An entity, a person, or a company that sells goods to consumers. In the pharmaceutical sector, this is the umbrella term for facilities that dispense/sell drugs (known as prescription-only medicines [POM] and Non-Prescription Medicines [NPM]) to patients, e.g. community pharmacies, other POM dispensaries, such as dispensing doctors, hospital pharmacies, pharmacy outlets, “medicine chests”, drugstores, supermarkets, etc.
Wholesaler	• An entity performing wholesale activities, i.e. procuring, holding, supplying or exporting drugs, apart from supplying drugs to the public
Distribution remuneration	• The payment of a health care provider (individual or organization) for the services provided. The services may be paid directly by the patient or by a third party payer. In the case of pharmaceutical distribution, wholesalers and pharmacies are remunerated by linear mark-ups, regressive margin schemes or, in the case of pharmacies, a fee-for-service remuneration
Price dependent remuneration	• Remuneration where the price of the product shapes the value or extent of the wholesale and/or pharmacy remuneration such as margins/mark-ups (regressive and fixed percentages)
Non-price dependent remuneration	• Distribution remuneration, which is not dependent on the price of the product and includes a fixed amount provided to the stakeholder for their services. Examples include a dispensing fee paid to the dispenser (usually pharmacy/pharmacist) to cover the costs of providing the service, professional services plus a reasonable profit
Margin	• The percentage of the selling price that is profit. In the case of pharmaceutical distribution, a wholesale or pharmacy margin is a price-dependent type of remuneration awarded to distribution actors, such as wholesalers and pharmacies, for performing their services. The wholesale margin is the gross profit of wholesalers, expressed as a percentage of the pharmacy purchasing price (wholesale price). The pharmacy margin is the gross profit of pharmacies expressed as a percentage of the pharmacy retail price
Mark-up	• The mark-up is the percentage of the purchasing price added on to arrive at the selling price. A mark-up is added on to the producer total cost of a good in order to create a profit $\circ$ The wholesale mark-up is the gross profit of wholesalers, expressed as a percentage add-on to the ex-factory price $\circ$ The pharmacy mark-up is the gross profit of pharmacies expressed as a percentage add-on to the wholesale price (or pharmacy purchasing price) $\circ$ Generally, mark-ups are regressive in nature (please see below for definition)
Regressive mark-up	• A mark-up whereby the size or value of the mark-up decreases as the price of the product increases. This may be on a sliding scale or applied in differential (discrete) steps according to threshold prices. Also called degressive and digressive
Fixed remuneration (flat fee or fixed percentage)	• A flat or set fee or a fixed percentage (known as linear mark up) which does not vary according to the cost of the item to which it is applied. Normally, this is a fixed fee that pharmacies are allowed to charge per prescribed item instead of or in addition to a percentage mark-up. The fee more accurately reflects the work involved in dispensing a prescription
Dispensing fee or charge	• An administrative charge that pharmacies are able to levy in addition to their standard remuneration (mark-up- or fee-related). This is typically levied on consumers rather than paid for by health insurance or the health system, unless it forms part of a contractual arrangement between health insurance/the health system and pharmacy association(s)

Source: The authors, drawn from [16, 22].

Our study involved two components: first, we identified, reviewed, synthesised, and analysed publicly available policy blueprints relating to distribution mark-ups, associated fees and charges, and taxes in order to capture pharmaceutical price build-up structures (from ex-factory to retail) in countries across Europe; the Middle East; Central, South, and East Asia; Latin America; and Africa that regulate the activities of the wholesale and retail distribution chain. Second, by using ex-factory price data across a number of product classes and the respective wholesale and pharmacy (i.e., retail community pharmacy) distribution mark-ups, associated fees and charges, and taxes, we used the information identified above to derive retail prices prevailing in the identified countries and for products in these product classes. By comparing ex-factory with retail prices, we were able to quantify the financial impact of distribution mark-ups, associated fees and charges, and taxes on health systems in the identified countries. In addition to wholesale and pharmacy mark-ups, we considered all practices relating to the application of fees and charges, which may typically apply at retail level (e.g., dispensing fees), as well as taxes on the consumption of prescription drugs (e.g., value added tax [VAT]).

### Data sources

#### Distribution mark-ups and taxation policies

We searched for available literature on distribution mark-up structure and related policies to identify scope countries for our analysis with a focus on settings where distribution mark-ups, associated fees, and taxes are regulated (data collection period: March to September 2021). We applied a number of search terms to identify both countries in scope and the associated policies on drug distribution and taxation. Search terms included “distribution mark-up(s)”, “distribution fees and charges”, “wholesale and retail mark ups”, “wholesale and retail fees and charges”, and “taxation of prescription drugs”. These terms were used to search and identify relevant peer reviewed literature on Medline and grey literature sources, particularly, information, policy documents, or reports from government and international organisation websites. The selection of countries in scope for our study was guided by the availability of relevant information and resulted in the identification of 35 countries across different regions (Box 2). Relevant information was obtained from sources including government websites, official publications, and reports, among others.

Box 2: Countries included in the study and sources for their distribution and taxation policies

**Table:** 

**Europe (26 countries)** [7, 23] Austria [24], Belgium [25], Bulgaria [26], Croatia [27–29], Czech Republic [30], Denmark [31], Estonia [32], Finland [33], France [34–36], Germany [37], Greece [38], Hungary [39], Ireland [40], Italy [41], Latvia [42, 43], Lithuania [44–46], Norway [47], Poland [48], Portugal [49], Romania [50], Slovakia [51], Slovenia [52], Spain [53], Switzerland [54], Sweden [55], and United Kingdom [56–59].
**The Middle East, Central, South, and East Asia (7 countries)** Jordan [60], Kazakhstan [61], Korea [62], Saudi Arabia [63], Turkey [64], United Arab Emirates [65, 66], and Vietnam [67].
**Latin America (1 country)** Colombia [68].
**Africa (1 country)** South Africa [69, 70].

We retrieved full texts of all papers, including policy documents, and captured relevant information on study objectives in a pre-defined data extraction template, including country name, year of publication of guidelines/policies, price build-up components and their structure by various distribution channels. We included the information as far as available and extracted wholesale and retail (pharmacy) mark-up information along with details on how they are applied (e.g., as fees or percentages), their type (e.g., regressive or flat), and any variations in their application (differences based on the type of products, distribution channel type, drug class, among others). Information was prioritized for drugs which are (a) reimbursed, (b) distributed through the retail channel/pharmacy, (c) patented, and (d) available as non-hospital drugs. If margins (rather than mark-ups) were the only available information source for any particular country (i.e., Italy, United Kingdom, Colombia, and Korea), these were converted in order to arrive at a homogeneous way of arriving at mark-ups (using mark-up = margin/[1 − margin]). Finally, information on dispensing fees and other relevant or applicable charges was also extracted.

#### Selection of ex-factory price categories for comparison

We extracted ex-factory prices using the IQVIA-Multinational Integrated Data Analysis System (IQVIA MIDAS^®^) database for the year 2020 [71, 72]. IQVIA MIDAS^®^, a commercial data platform assessing worldwide healthcare markets, provides estimated product volumes, trends, and market shares through retail and non-retail channels across various therapeutic classes based on the European Pharmaceutical Market Research Association (EphMRA)/Intellus Anatomical Therapeutic Chemical (ATC) classification. The four-level classification primarily categorizes products based on indication (level 1), therapeutic substance group (level 2), and anatomical system (level 3). The fourth level provides further detail, including formulation, chemical description, and mode of action, among others [73].

In order to quantify the financial impact of distribution mark-ups, associated fees and charges, and taxes on health systems in the study countries, we translated the available ex-factory prices into “pharmacy retail prices” for three product classes by applying mark-up and taxation information. The three product classes were selected from a list of product classes with high sales and represented “high-”, “medium-” and “low-price” points respectively, as we endeavoured to assess the impact of distribution policies and taxes across a range of product categories with different price levels; this was deemed essential considering that distribution policies are very frequently regressive in nature, such that high-price products attract low relative mark-ups and vice versa [11]. In order to select the three product class price points, we proceeded as follows: first, we used sales data at ex-factory level for ATC1 classes in order to identify the seven product classes with the highest sales (in billion US$) that contributed to more than 80% of global market sales, which included ATC1 classes “L” (antineoplastic and immuno-modulating agents), “A” (alimentary tract and metabolism), “N” (nervous system), “J” (general anti-infectives systemic), “C” (cardiovascular system), “R” (respiratory system), and “B” (blood and blood-forming organs) (Appendix Table 1); the seven ATC1 classes contained 57 ATC2 and 203 ATC3 sub-categories based on the product class distribution of data. We ranked the 203 ATC3 sub-categories by sales and identified the ten therapeutic sub-classes with the highest global sales (in billion US$) (Appendix Table 2). For each of the ten therapeutic sub-categories, we calculated the weighted average ex-factory prices by dividing the total sales for the respective ATC3 category by total volume across the 35 study countries. We used information per pack as a metric of volume because margins/mark-ups are applied at per pack level. We used weighted ex-factory prices by using country volume weights in order to arrive at a more representative price index (known as Paasche price index) as a price proxy for the therapeutic class. This was done because using a simple average price may be misleading. The Paasche price index takes into consideration consumption patterns by using current quantities (current weightings) and is not upward biased in terms of price increases. The weighted average ex-factory prices ranged from US$2 for “Non-narcotics and Anti-pyretics” to US$1355 for “Antineoplastic Monoclonal Antibodies” (Appendix Table 2).

Finally, we categorized these ten therapeutic classes based on the calculated ex-factory price as “high-priced” (with an ex-factory price more than US$100), “medium-priced” (with an ex-factory price between US$10 and US$99), and “low-priced” drugs (with an ex-factory price of < US$10). We selected the product class with the highest weighted average price from each of these three categories to further analyse the impact of distribution mark-ups and taxes on the retail price. Therefore, for the “high-”, “medium-”, and “low-priced” drug categories, “Monoclonal Antibody Antineoplastics” (L1G, US$1355), “GLP-1 agonist anti-diabetics” (A10S, US$96), and “anti-epileptics” (N3A, US$8), respectively, were selected (referred to as scenario analysis). The three price points represent the three price categories in a comprehensive manner and include different types of drugs i.e., innovative as well as generic medicines. Box 3 provides an overview of the selection process for the three product class price categories.

Box 3: Overview of selection of three price categories for evaluating the impact of mark-up structure on retail prices

**Table:** 


*Ex-factory prices were weighted based on sales volume for each product class **High-priced drugs included antineoplastic monoclonal antibodies, anti-TNF products, and protein kinase inhibitors; medium-priced drugs included GLP-1 antagonists (anti-diabetics), direct factor XA inhibitors, and human insulin + analogues; and low-priced drugs included anti-epileptics, cholesterol and triglyceride regulators, anti-ulcerants, and non-narcotic analgesics ATC: Anatomical Therapeutic Chemicals classification system; MIDAS: Multinational Integrated Data Analysis System; WHO: World Health Organization

Mark-up ranges, fixed fees, and tax information from national policy blueprints/guidelines were available in local currencies. In order to compare these across countries, we converted each of the selected scenario price points (US$8, US$96, and US$1355) to local currencies, following which, we applied rates for mark-ups and taxes for the estimation of the retail price for individual countries. Finally, we converted these estimates back to US$ to enable cross-country comparisons by using 2020 exchange rates based on the International Monetary Fund’s (IMF) currency conversion calculator [74].

### Analysis

We conducted a descriptive comparative analysis to synthesise and present the extracted information regarding the distribution channel components and taxes across countries. Based on this, we drew general conclusions on the structure of distribution remuneration strategies and taxation, and discussed key trends in wholesale and retail distribution and taxation across settings that are regulated.

In order to evaluate the impact of mark-up and tax structures on the cost of prescription drugs, we conducted scenario analyses to assess retail price levels in all scope countries considering the weighted average ex-factory price for the three selected price categories (Appendix Table 2). Results from the scenario analysis are graphically presented where the selected three price points are depicted as baseline and the applicable distribution mark-ups, fees, and taxes are added on to derive retail prices in each setting (absolute value in US$).

To further evaluate the impact of mark-ups and taxes on the remaining price points from the three price categories and assess the generalizability of findings from the scenario analyses, we conducted sensitivity analyses using the “anti-TNF products” (L4B), “protein kinase inhibitor antineoplastics” (L1H), “direct factor XA inhibitors” (B1F), “human insulins and analogs” (A10C), “cholesterol & triglycerides regulating preparations” (C10A), “anti-ulcerants” (A2B), and “non-narcotics and antipyretics” (N2B) based on the identified product classes. The impact results from the sensitivity analysis are shown in Appendix Figures 4–10.

## Results

### Taxonomy of and evidence on distribution and taxation policies

The majority of selected countries regulate both parts of the distribution chain (*n* = 27, 77%) and only few regulate either wholesaling (*n* = 1; Colombia) or retailing (*n* = 7), but leave one part unregulated (UK, where the wholesale margin is reported to be no higher than 12.5%) or unregulated and subject to negotiation with competent health authorities (Denmark, Finland, Norway, Sweden, Korea, and Vietnam).

In general, the distribution remuneration can be categorized as price-dependent remuneration (i.e., margins/mark-ups) and non-price dependent remuneration (i.e., dispensing fee) as described in Box 1. To further evaluate how they are applied in routine practice, we classified wholesale and retail/pharmacy remuneration into three types. The first type is a r*egressive mark-up structure (price-dependent)*, which could take the form of (a) regressive percentages, or (b) regressive fees, or (c) a combination of regressive percentages and fees, whereby both percentages and fees (in relative % terms) are used in declining order as part of a regressive scheme to remunerate the distribution chain; 11 countries (31%) used this form of remuneration in wholesale and 15 countries (43%) used the same in retail distribution. The second type took the form of *regressive mark-up with capping and single fixed (flat) fee beyond the cap (price-dependent as concerns the regressive mark up structure and non-price dependent for the part that relates to fixed fees)* and consisted of countries that use a regressive mark-up up to a ceiling, beyond which prescription drugs are paid for by a fixed fee; five countries (14%) and nine countries (26%) use this form of remuneration in wholesale and retail distribution, respectively. The third type was *fixed remuneration either on a percentage basis or on a fee basis (non-price dependent)*; 19 countries (54%) used this form of remuneration in wholesale and 10 countries (29%) used the same in retail distribution. For retail distribution only, seven countries (Belgium, Denmark, Finland, France, Germany, Norway, and the UK) apply modest *dispensing fees (non-price dependent)*. It is important to note here that for a few countries (Italy, United Kingdom, Colombia, and Korea), remuneration information was available in the form of margins rather than mark-ups. However, in order to ensure comparability across the study countries, they are presented as mark-ups as the inherent nature of how they are applied (fixed or regressive), does not change. The mark-up regulations across the study countries varied based on the type of product, distribution channel type, and reimbursement status. Table 1 summarizes available information on wholesale and pharmacy remuneration structures, the applicable ranges, and VAT rates across the 35 countries for prescription drugs in a descriptive manner. The information provides a fair indication of mark-up and taxation situation in the study countries and reflects the year information was available.

**Table 1:** Overview of distribution mark-up/margin structure and taxation for reimbursed pharmaceuticals in 35 countries, 2021*

Country (Publication year)**	Wholesale mark-up/margin information				Pharmacy mark-up/margin information				Dispensing fee or charge	Regressive categories (*n*, wholesale/pharmacy)	VAT (%)	Mark-up/margin information***
	Regressive type			Fixed remuneration type (flat fee or %)	Regressive type			Fixed remuneration type (flat fee or %)				
	Percent (%)	Fee	Combination of % & fee		Percent (%)	Fee	Combination of % & fee					
AT^a^ (2019)	–	–	**✓ (₸)**	**✓ (Fee)**	–	–	**✓ (₸)**	**✓ (Fee)**	–	10/19	10%	*Wholesale*: 15% to 7% with flat fee [cap] of €23.74 [US$28.21] on ex-factory price above €339.15 [US$403.05] *Pharmacy*: 17.5% to 9% with flat fee [cap] of €30.52 [US$36.27] on ex-factory price above €339.15 [US$403.05]
BE^b^ (2018)	–	–	**✓**	–	–	–	**✓**	–	€4.33 (US$5.15) per reimbursed pack	4/2	6%	*Wholesale*: 15% (minimum €0.35 [US$0.42]) to “0.9% + additional €2 [US$2.38]” *Pharmacy*: Ex-factory price < €60 (< US$71.30): 6.55%; Ex-factory price ≥ €60 (≥ US$71.30): “2.3% + additional €4.188 (US$4.98)”
BG (2020)	**✓ (₸)**	–	–	**✓ (Fee)**	**✓ (₸)**	–	–	**✓ (Fee)**	–	3/3	20%	*Wholesale*: 7% to 4% with flat fee [cap] of BGN 10 (US$6.09) on ex-factory price above BGN 30 [US$18.26] *Pharmacy*: 20% to 16% with flat fee [cap] of BGN 25 [US$15.22] on ex-factory price above BGN 30 [US$18.26]
HR^b^ (2019)	–	–	–	**✓ (%)**	–	–	–	**✓ (Fee)**	–	–/–	5%	*Wholesale*: Maximum of 8.5% *Pharmacy*: €0.92 (US$1.09) per prescription
CZ (2018)	–	–	**✓**	–	–	–	**✓**	–	–	8/8	10%	*Wholesale* and * Pharmacy*: A total of 37% to “2% + additional 858 CZK (US$40.02)” mark-up. Proportion of share between WS and pharmacy is not known
DK (2021)	–	–	–	**✓ (%)**	–	–	–	**✓ (%)**	DKK 8 (US$1.28) without VAT	–/–	25%	*Wholesale*: ~ 6–7% (private negotiations, unregulated) *Pharmacy*: 7.6%
EE^c^ (2005)	**✓ (₸)**	–	–	**✓ (Fee)**	–	–	**✓ (₸)**	**✓ (Fee)**	–	6/8	9%	*Wholesale*: 20% to 3% with flat fee [cap] of €6.40 [US$7.61] on ex-factory price above €213.33 [US$253.52] *Pharmacy*: For ex-factory price < €0.64 (US$0.76), a fixed fee of €0.38 (US$0.45) is applicable; For ex-factory price €0.64 (US$0.76) to €1.28 (US$1.52): 40% + €0.38 (US$0.45); For ex-factory price between €1.28 (US$1.52) to €44.74 (US$53.17): 35% to 15%; For ex-factory price above €44.74 [US$53.17]: flat fee [cap] of €5.11 [US$6.07]
FI (2020)	–	–	–	**✓ (%)**	–	–	**✓**	–	€2.17 (US$2.58)	–/5	10%	*Wholesale*: ~ 3% (private negotiations, unregulated) *Pharmacy*: 45% to “10% + additional fee of €36.65 (US$43.55)”
FR (2020)	–	–	–	**✓ (%) and (₸) (Fee)**	**✓**	–		–	For reimbursed products additional remuneration per pack of €1.02 (US$1.21)	–/5	2.1%	*Wholesale*: Ex-factory price ≤ €468.97 (≤ US$557.32): 6.93% (minimum of €0.30 [US$0.36] and a ceiling of €32.50 [US$38.62]); Ex-factory price > €468.97 (> US$557.32): €32.50 (US$38.62) *Pharmacy*: 10% to 5%
DE (2021)	–	–	–	**✓ (%) and (₸) (Fee)**	–	–	–	**✓ (%)**^**k**^	€8.35 (US$9.92) with additional surcharge of €0.21 (US$0.25)	**–**/**–**	19%	*Wholesale*: 3.15% + additional €0.7 (US$0.83) with a capping of €37.8 (US$44.92) *Pharmacy*: 3% plus fees and surcharges
GR^b^ (2019)	**✓**	–	–	–	**✓**	–	–	–	–	2/21	6%	*Wholesale*: Ex-factory price ≤ €200 (≤ US$237.68): 4.9%; Ex-factory price > €200 (> US$237.68): 1.5% *Pharmacy*: 30% to 2%
HU (2007)	–	–	**✓**	–	–	–	**✓ (₸)**	**✓ (Fee)**	–	4/8	5%	*Wholesale*: 8% for Below 500 HUF (US$1.69) ex-factory price; 501 HUF (US$1.7) to 1,000 HUF (US$3.39) ex-factory price: 6.5% with a minimum of 40 HUF (US$0.14); 1,001 HUF (US$3.39) to 2,000 HUF (US$6.78) ex-factory price: 5% with a minimum of 65 HUF; > 2,000 HUF (> US$6.78) ex-factory price: 4.4% with a minimum of 100 HUF (US$0.34) *Pharmacy*: 27% to 15% (flat fee [cap] of 990 HUF [US$3.35] on ex-factory price above 5,500 HUF [US$18.63])
IE^d^ (2021)	–	–	–	**✓ (%)**	–	–	–	**✓ (Fee)**	–	–/–	23%	*Wholesale*: 8% fixed for items on the CDS *Pharmacy*: Applied as pharmacy fee, i.e. dispensing fee; varies based on scheme employed. €5.48 (US$6.51) per item per month for items on the CDS
IT (2019)	–	–	–	**✓ (%)**	–	–	–	**✓ (%)**	–	–/–	10%	*Wholesale*: Fixed as wholesaler margin 3% on Public price without VAT *Pharmacy*: Fixed as pharmacy margin 30.35% on Public price without VAT
LV^b^ (2015)	**✓**	–	–	–	–	–	**✓**	–	–	9/9	12%	*Wholesale*: 10% to 1% *Pharmacy*: 30% to 5% (Capping: Fixed fee of €6.05 [US$7.19] above ex-factory price of €71.14 [US$84.54])
LT^b^ (2019)	–	**✓ (₸)**	–	**✓ (Fee)**	–	**✓ (₸)**	–	**✓ (Fee)**	–	3/3	5%	*Wholesale*: €0.51 (US$0.61) to a maximum of €5.79 (US$6.88; capped) *Pharmacy*: €1 (US$1.19) to a maximum of €14.48 (US$17.21; capped)
NO^e^ (2019)	–	–	–	**✓ (%)**	–	–	–	**✓ (%)**	29 NOK (US$3.39) per pack or additional 19 NOK (US$2.22) if it is classified as A/B (addictive)	–/–	25%	*Wholesale*: Private negotiations, unregulated (~ 6–7%) *Pharmacy*: 2%
PL^f^ (2012)	–	–	–	**✓ (%)**	**✓**	–	**–**	–	–	–/10	8%	*Wholesale*: 5% *Pharmacy*: 40% to 1.2%
PT (2015)	–	–	**✓**	–	–	–	**✓**	–	–	6/6	6.42%	*Wholesale*: “2.2% + additional €0.25 (US$0.30)” to “1.2% + additional €3.68 (US$4.37)” *Pharmacy*: “5.6% + additional €0.63 (US$0.75)” to “2.7% + additional €8.28 (US$9.84)”
RO (2017)	**✓ (₸)**	–	–	**✓ (Fee)**	**✓ (₸)**	–	–	**✓ (Fee)**	–	4/5	9%	*Wholesale*: 14% to 10% with flat fee [cap] of RON30 [US$7.24] on ex-factory price above RON300 [US$72.4] *Pharmacy*: 24% to 12% with flat fee [cap] of RON35 [US$8.45] on ex-factory price above RON300 [US$72.4]
SK (2009)	–	–	**✓**	–	–	–	**✓**	–	–	11/11	10%	*Wholesale*: 14% to “1.8% + additional €14.78 [US$17.56]” *Pharmacy*: 32.9% to “4.2% + additional fee of €34.48 [US$40.98]”
SI (2015)	–	–	**–**	**✓ (%) (₸) (Fee)**	–	–	**–**	**✓ (%) (₸) (Fee)**	–	–/–	9.5%	*Wholesale* and * Pharmacy*: 1.1% of ex-factory price (ranges from €0.5 [US$0.59] to €27.5 [US$32.68]). Proportion of share between WS and pharmacy is not known
ES (2008)	–	–	–	**✓ (%) (₸) (Fee)**	–	–	**✓ (₸)**	**✓ (Fee)**	–	–/4	4%	*Wholesale*: Ex-factory price ≤ €91.64 (≤ US$108.9): 7.6%; Ex-factory price > €91.64 (> US$108.9): A fixed fee capped at €7.54 (US$8.96) *Pharmacy*: 27.9% to a maximum of €48.37 (US$57.48; capped) on ex-factory price above €500 (US$594.2)
CH (2016)	**✓ (₸)**	–	–	–	–	–	**✓ (%) (₸) (Fee)**	**✓ (Fee)**	–	3/6	2.5%	*Wholesale* and * Pharmacy*: Data available for overall distribution channel; ranges from 12 to 7% (Capping: Zero mark-up above ex-factory price of 2,570 CHF [US$2784.7]) Additionally, a regressive fee for retailers is applicable from 4 CHF (US$4.33) for ex-factory price < 5 CHF (< US$5.42) to maximum of 240 CHF (US$260.05) for ex-factory price > 2,570 CHF (> US$2979.74)
SE^g^ (2017)	–	–	–	**✓ (%)**	–	–	**✓ (₸)**	**✓ (Fee)**	–	–/4	0%	*Wholesale*: ~ 2- 3% (private negotiations, unregulated) *Pharmacy*: “20% + additional fee of 30.5 SEK (US$3.58)” to “2% + additional fee of 46.25 SEK (US$5.43)”. Flat fee (cap) of 1,046.25 SEK (US$122.94) on ex-factory price above 50,000 SEK (US$5875.23)
GB (2010)	–	–	–	**✓ (%)**	–	–	–	**✓ (%)**	£1.29 (US$1.79) per item for all prescriptions < £100 (< US$138.63) and 2% for > £100 (> US$138.63)	–/–	0%	*Wholesale* and * Pharmacy*: An overall margin of 12.5% (nominal to wholesale distribution), of which a significant proportion (can be 10% of the 12.5%) is offered to pharmacy as a discount *Pharmacy*: Subject to pharmacy contract based on volume of dispensing and other services provided; discounts received from wholesalers and direct purchases from manufacturers, esp. for generic drugs
JO^h^ (2020)	–	–	–	**✓ (%)**	–	–	–	**✓ (%) (₸) (Fee)**	–	–/–	4%	*Wholesale*: 23.4% *Pharmacy*: For drugs with ex-factory price < 150 JOD (< US$211.57): 26%; 15 JOD (US$21.16) for drugs with ex-factory price > 150 JOD (> US$211.57)
KZ (2020)	**✓**	–	–	–	**✓**	–	–	–	–	11/11	0%	*Wholesale*: 21% to 10% *Pharmacy*: 55% to 10%
KR^i^ (2018)	–	–	–	**✓ (%)**	–	–	–	–	–	–/–	10%	*Wholesale*: Fixed percentage margin agreed upon in negotiations but thought to range between ~ 7.5%—8.6% (private negotiations, unregulated) *Pharmacy*: Pharmacy margin or dispensing fee not permitted. Annual service fee received
SA (2019)	**✓**	–	–	–	**✓**	–	–	–	–	2/3	0%	*Wholesale*: 15% to 10% *Pharmacy*: 20% to 10%
TR (2015)	**✓**	–	–	–	**✓**	–	–	–	–	5/3	8%	*Wholesale*: 9% to 2% *Pharmacy*: 25% to 12%
AE (2020)	–	–	–	**✓ (%)**	**✓**	–	–	–	–	–/3	0%	*Wholesale*: 15% *Pharmacy*: 28% to 20%
VN (2011)	–	–	–	**✓ (%)**	**✓**	–	–	–	–	–/5	5%	*Wholesale*: Fixed percentage agreed upon in negotiations but thought to range between ~ 3–5% (private negotiations, unregulated) *Pharmacy*: 15% to 2%
CO^j^ (2021)	–	–	–	**✓ (%)**	–	–	–	**✓ (%)**	–	–/–	0%	*Wholesale*: 7% margin *Pharmacy*: Fixed percentage agreed upon in negotiations but thought to range between ~ 15 to 20% margin (private negotiations, unregulated) Also, for regulated price drugs, an additional margin of 3.5% (if maximum sale price > 1,000,000 COP [> US$266.19]) to 7% (if maximum sale price < 1,000,000 COP [< US$266.19]) is granted to Health Service Provider Institutions
ZA (2021)	–	–	–	**✓ (%)**		–	**✓**^**l**^	–	–	4/–	15%	*Wholesale*: Varies ~ 7–20% by company and product type *Pharmacy*: Applies to SEP only If SEP < R113.71 (< US$7.96): R15.95 (US$1.12) + 46.0% of the SEP; If SEP < R303.31 (< US$21.22): R29.07 (US$2.03) + 33.0% of the SEP; If SEP < R1061.62 (< US$74.28): R82.77 (US$5.79) + 15.0% of the SEP; If SEP > R1061.62 (> US$74.72): R190.68 (US$13.34) + 5.0% of the SEP

Country abbreviations: *AE* United Arab Emirates; *AT* Austria; *BE* Belgium; *BG* Bulgaria; *CSE* Central, South, and East Asia; *CH* Switzerland; *CO* Colombia; *CZ* Czech Republic; *DE* Germany;

*DK* Denmark; *EE* Estonia; *ES* Spain; *FI* Finland; *FR* France; *GB* United Kingdom; *GR* Greece; *HR* Croatia; *HU* Hungary; *IE* Ireland; *IT* Italy; *JO* Jordan; *KR* Korea; *KZ* Kazakhstan; *LATAM* Latin America; *LT* Lithuania; *LV* Latvia; *NO* Norway; *PL* Poland; *PT* Portugal; *RO* Romania; *SA* Saudi Arabia; *SE* Sweden; *SI* Slovenia; *SK* Slovakia; *TR* Turkey; *VN* Vietnam; *ZA* South Africa. Currency acronyms and other terms: *BGN* Bulgarian Lev; *CDS* Community Drug Scheme; *CHF* Swiss Franc; *COP* Colombian Peso; *CZK* Czech Koruna; *DKK* Danish Krone; *HT* High Tech Drug Agreements; *HUF* Hungarian Forint; *JOD* Jordanian Dinar; *NOK* Norwegian Krone; *R* South African Rand; *RON* Romanian Leu; *SEK* Swedish Krona; *SEP* Single Exit Price; *VAT* Value Added Tax

**“₸”** represents capping; Fee: Mark-up applied as a fixed fee; %: Mark-up applied as a percentage

*Details on remuneration and applicable fees are provided in local currency based on country-specific regulations. Local currency figures have also been converted the US$ to enable cross-country comparability. The 2020 average local currency vs. the US$ exchange rate has been used for this purpose based on the IMF currency conversion calculator [25]

**Year in brackets represents the publication year of the latest available data on distribution remuneration for the respective countries

***Information presented in the table represents mark-ups unless otherwise specified, i.e. for Italy, UK, Korea, and Colombia, where margin information was reported as mentioned in the table

Other notes: Country-specific variation in the mark-up information is provided below. **Text in bold** indicates mark-up information reported in table and prioritized for the analysis:

^a^Wholesale mark-up: Varies by **reimbursed** and non-reimbursed product type; pharmacy mark-up: Varies by customer type (privileged vs. private)

^b^Wholesale and pharmacy mark-up: Vary by **reimbursed** and non-reimbursed product type

^c^Wholesale mark-up and pharmacy mark-up: Vary by **retail **and hospital channel type

^d^Wholesale mark-up: Varies by reimbursement schemes (**CDS**, HT, and hospital drugs), product type (**biologics** and non-biologics), and exclusivity (**patented** and off-patent). Moreover, mandatory rebates are applicable in Ireland (5.5% for patented exclusive drugs in CDS). However, these are not accounted for in our analysis assuming this cost as a hidden cost to the overall healthcare system

^e^Wholesale mark-up: Assumed as 6–7% for the calculations based on Denmark mark-ups

^f^Wholesale and pharmacy mark-up: Vary by **reimbursed** (**open market** vs. drug program channel type) and non-reimbursed product type

^g^Wholesale and pharmacy mark-up: Vary for products with generic competition and **without generic competition**

^h^Pharmacy mark-up: Varies for hospital and **non-hospital** drugs

^i^Wholesale mark-up: Varies by **reimbursed** and non-reimbursed product type as well as by the company type (**domestic** vs. multi-national)

^j^Pharmacy mark-up: Varies by channel type (distributed through **drug store/pharmacy** vs. hospital/institutions)

^k^For detailed account on dispensing fee and surcharges, please refer to this link: https://www.gesetze-im-internet.de/ampreisv/__3.html

^l^For detailed information on pharmacy mark-up based on SEP, please refer to this link: https://www.greengazette.co.za/notices/medicines-and-related-substances-act-101-1965-regulations-relating-to-a-transparent-pricing-system-for-medicines-and-schedules-substances-dispensing-fee-for-pharmacists_20200619-GGN-43447-00679.pdf. Source: The authors from the literature

#### Wholesale mark-up structure

Among the countries with regressive mark-up structure to remunerate wholesale distribution described above (*n* = 11), six countries have a percentage type regressive mark-up (Greece, Latvia, Switzerland, Kazakhstan, Saudi Arabia, and Turkey), whereas five have a combination of percentage and fixed fee (Belgium, Czech Republic, Hungary, Portugal, and Slovakia).

Among the countries with fixed mark-up structure (*n* = 19), a fixed percentage type mark-up was applicable. However, in four of these 19 countries (France, Germany, Slovenia, and Spain), mark-ups are applied as a single fixed fee beyond a certain price point. In the Czech Republic, a joint mark-up exists between the wholesale and retail channel, which is shared by both parties (proportion of share for each side is not known), while in Switzerland, information was available for the entire distribution channel without specific mark-up split between wholesale and pharmacy (Table 1).

Finally, five countries (Austria, Bulgaria, Estonia, Lithuania, and Romania) had a combination of regressive mark-up with fixed remuneration (flat fee). Mark-ups are often applied as increasing fixed fees with an increase in ex-factory price ranges in Lithuania, however, the overall nature of the mark-up structure is regressive when quantified in percentage terms.

Overall, for 16 countries that apply a regressive mark-up structure, the number of regressive categories range from two (Greece and Saudi Arabia) to 11 (Kazakhstan and Slovakia) (Table 1).

#### Pharmacy mark-up structure

Among the countries with a regressive mark-up structure for retail distribution described above (*n* = 15), eight countries have a percentage type regressive mark-up (France, Greece, Kazakhstan, Poland, Saudi Arabia, Turkey, UAE, and Vietnam), while seven have a combination of percentage and fixed fee (Belgium, Czech Republic, Finland, Latvia, Portugal, Slovakia, and South Africa).

Among the countries with a fixed remuneration structure (*n* = 10), eight have a fixed percentage type mark-up (Colombia, Denmark, Germany, Italy, Jordan, Norway, Slovenia, and the UK) and two implement fixed fee type mark-ups (Croatia and Ireland). In Slovenia and Jordan, pharmacy mark-ups are applied as a single fixed fee beyond a certain price point. Specifically, in Ireland, the fixed fee (levied as a pharmacy fee) is based on a sliding scale and is recommended to be an average of €5.48 (US$6.51) per item per month for drugs on the community drug schemes (CDS) as per the National Centre for Pharmacoeconomics (NCP) guidelines [40]. In the UK, a Single Activity Fee (SAF) of £1.29 (US$1.79) is charged as fixed remuneration for all products as an additional mark-up component in the price-to-public determination and a fee equivalent to 2% of net ingredient cost payable on all prescriptions over £100 (US$138.63) [57]. This is, however, an understatement of UK pharmacy remuneration, as (a) they are paid on the basis of volume of services provided as outlined in the Pharmacy Contract [59] and (b) receive discounts from suppliers of certain types of prescription drugs and a proportion of that discount, currently at 8%, is withheld by the NHS as ‘discount deduction’ at the point of settling invoices with community pharmacies and pharmacy chains; the discounts form part of the pharmacies’ income [58]. In Colombia, for price-regulated drugs, an additional margin is granted to Health Service Provider Institutions (IPS) to recognize the value they add to the drug distribution chain. For drugs with a maximum sale price less than or equal to COP 1,000,000 (US$266.19), they may add a percentage up to 7%. For drugs with a maximum sale price greater than COP 1,000,000 (US$266.19), they may add a percentage of up to 3.5% [68].

There are nine countries with a regressive mark-up structure followed by a single fixed fee beyond a certain ex-factory price level (Austria, Bulgaria, Estonia, Hungary, Lithuania, Romania, Spain, Switzerland, and Sweden). In Lithuania, similar to the wholesale mark-up structure, pharmacy mark-ups are applied as increasing fixed fees with an increase in ex-factory price ranges. In Switzerland, as noted above, discrete information on pharmacy and wholesale mark-up was not available, however, an additional fee is provided to retailer that ranges from CHF 4 (US$4.33) per pack with an ex-factory price of < CHF 5 (US$5.42) to a maximum of CHF 240 (US$260.05) for drugs with an ex-factory price > CHF 2570 (US$2784.7). In South Africa, a pharmacy mark-up applies to the ‘single exit price’ (SEP) and ranges from R15.95 (US$1.12) + 46.0% of the SEP (if the SEP is < R113.71 [US$7.96]) to R190.68 (US$13.34) + 5.0% of the SEP (if the SEP is > R1061.62 [US$74.28]) [70]. Overall, for the countries with regressive pharmacy mark-up structure (*n* = 24), the number of regressive categories ranges from two (Belgium) to 21 (Greece) (Table 1).

Finally, in Korea, pharmacies cannot charge a mark-up for prescription drugs covered by the National Health Insurance (NHI) and receive an annual service fee instead [62].

#### Taxation (VAT)

**Fig. 1:**
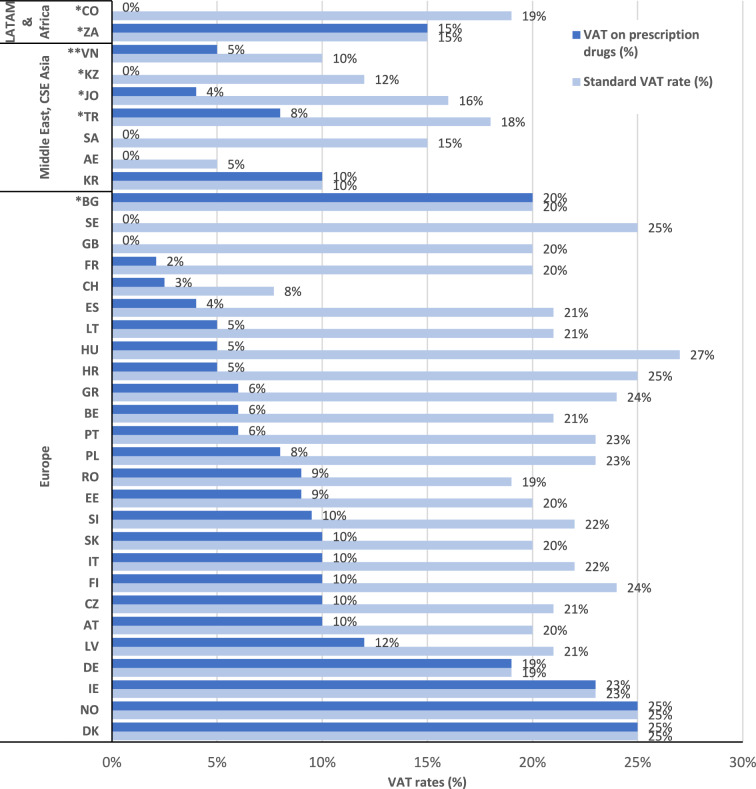
A comparative overview of VAT rates for prescription pharmaceuticals and other commodities (standard VAT) across the 35 countries. Abbreviations: *AE* United Arab Emirates; *AT* Austria;* BE* Belgium; *BG* Bulgaria; *CSE* Central, South, and East Asia; *CH* Switzerland; *CO* Colombia; *CZ* Czech Republic; *DE* Germany; *DK* Denmark; *EE* Estonia; *ES* Spain; *FI* Finland; *FR* France; *GB* United Kingdom; *GR* Greece; *HR* Croatia; *HU* Hungary; *IE* Ireland; *IT* Italy; *JO* Jordan; *KR* Korea; *KZ* Kazakhstan; *LATAM* Latin America; *LT* Lithuania; *LV* Latvia; *NO* Norway; *PL* Poland; *PT* Portugal; *RO* Romania; *SA* Saudi Arabia; *SE* Sweden; *SI* Slovenia; *SK* Slovakia; *TR* Turkey; *VN* Vietnam; *ZA* South Africa. *Upper middle-income countries; **Lower middle-income countries. All other markets are high income countries according to the World Bank classification [75]; VAT rates may vary based on reimbursement status or type of drug, e.g., France: 2.1% for reimbursable drugs and 10% for non-reimbursable drugs; Ireland: 0% for oral drugs and 23% for other drugs; Lithuania: 5% for reimbursable drugs and 21% for non-reimbursable drugs. Source. The authors from EFPIA [23]; OECD and other sources [16, 76–81]

Overall, taxes on prescription drugs are applicable in 29 of the 35 countries (83% of the sample) (Fig. 1), with Norway and Denmark applying the highest rate (25% on the retail price). In 7 of the 29 countries that apply VAT (Denmark, Norway, Ireland, Germany, Bulgaria, Korea, and South Africa), the standard VAT rate is the same for both prescription drugs and other commodities, while in the remaining 22 countries (63%) that apply VAT on prescription drugs, the applicable rate is lower vs. other commodities. In the remaining six countries (17%) the applicable VAT is zero percent (UK, Sweden, Kazakhstan, Saudi Arabia, UAE, and Colombia).

### Price component analysis

**Fig. 2:**
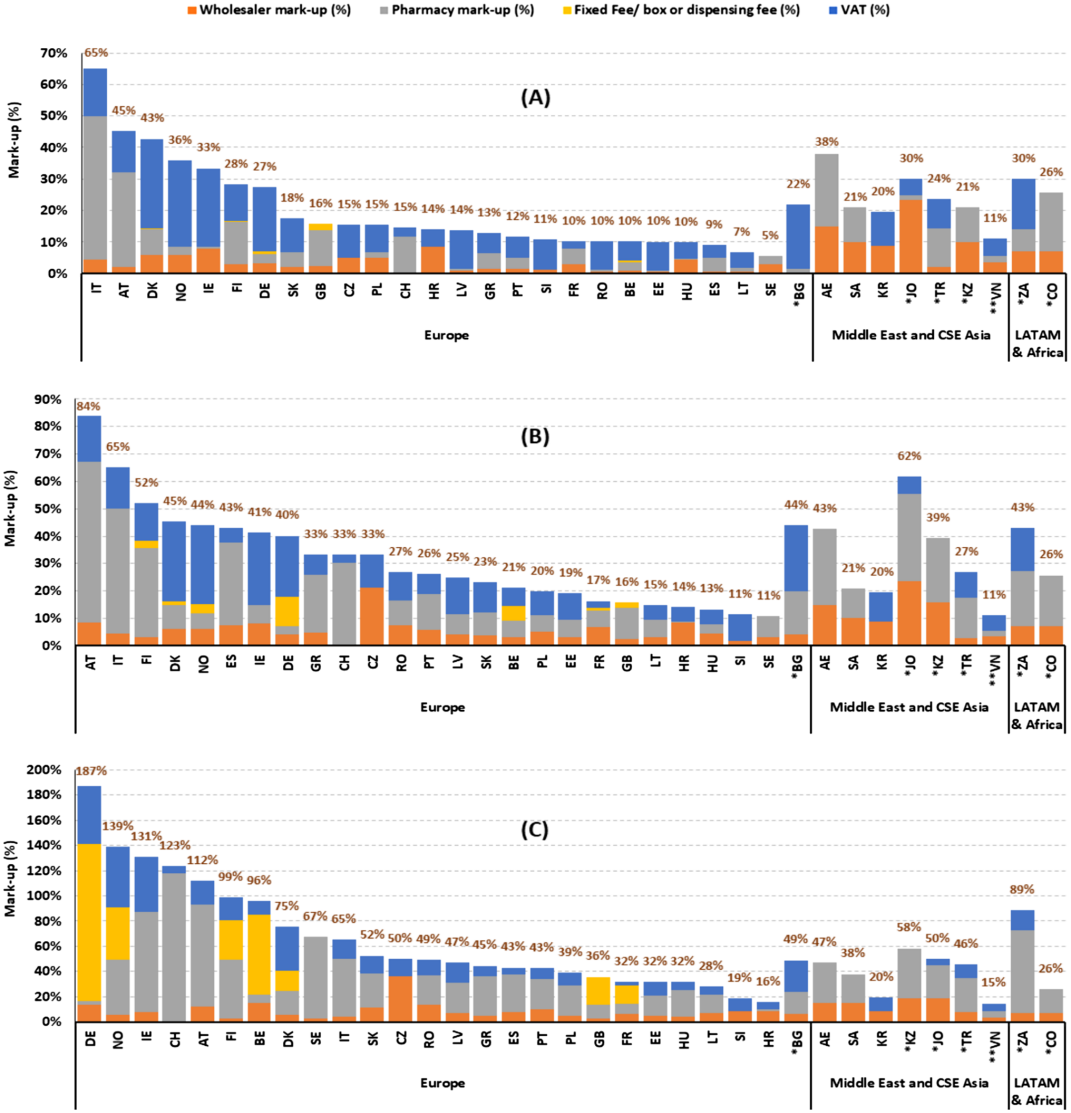
Price components as percentage of the ex-factory price for prescription drugs. Panel (**A**) Monoclonal Antibodies (ex-factory price: US$1355), Panel (**B**) GLP-1 Agonists (ex-factory price: US$96), and Panel (**C**) Anti-epileptics (ex-factory price: US$8). Abbreviations: *AE* United Arab Emirates; *AT* Austria; *BE* Belgium; *BG* Bulgaria; *CH* Switzerland; *CO* Colombia; *CZ* Czech Republic; *DE* Germany; *DK* Denmark; *EE* Estonia; *ES* Spain; *FI* Finland; *FR* France; *GB* United Kingdom; *GR* Greece; *HR* Croatia; *HU* Hungary; *IE* Ireland; *IT* Italy; *JO* Jordan; *KR* Korea; *KZ* Kazakhstan; *LT* Lithuania; *LV* Latvia; *NO* Norway; *PL* Poland; *PT* Portugal; *RO* Romania; *SA* Saudi Arabia; *SE* Sweden; *SI* Slovenia; *SK* Slovakia; *TR* Turkey; *VN* Vietnam; *ZA* South Africa. *Upper middle-income countries; **Lower middle-income countries. All other markets are high income countries according to the World Bank classification [75]; For CZ and SI, a combined mark-up information was present for wholesale and pharmacy. In the figure, total mark-up is illustrated as wholesale mark-up. Source. The authors based on IQVIA MIDAS^®^ data

The price component analysis (scenario analysis) revealed that the combination of wholesale and pharmacy mark-ups along with VAT varies considerably across both the study countries and the three price point categories. Figure 2 presents the impact of mark-ups and taxes on the prices of the three drug categories selected: (A) monoclonal antibodies (“high” ex-factory price: US$1355), (B) GLP-1 agonists (“medium” ex-factory price: US$96), and (C) anti-epileptics (“low” ex-factory price: US$8).

For the high-price drug category, the countries with the highest mark-up plus tax cost included Italy and Austria, where the mark-ups plus taxes accounted for 65% and 45% of the ex-factory price of the drug, respectively. The pharmacy mark-up was the major contributor to this cost, accounting for 46% and 30% of the ex-factory price, respectively. Denmark (43%), UAE (38%), and Norway (36%) were the other countries in the top five countries with highest mark-ups plus tax cost. The country with the lowest mark-up cost was Sweden, where mark-ups along with taxes constituted only 5% of the ex-factory price (Fig. 2). Variations in the amplitude and extent of distribution mark-up and tax policies are also significant among neighbouring countries, reflecting different philosophies in distribution and/or taxation policies: for example, average distribution costs are 43% in Denmark, but only 5% in Sweden; similarly, VAT on prescription drugs is a fixed 25% (amounting to 29% of the ex-factory price) in Denmark, but is 0% in Sweden.

For the medium-price drug category, the proportion of mark-ups plus tax cost to the ex-factory price ranged from 11% (Slovenia, Sweden, and Vietnam) to 84% for Austria. Pharmacy mark-ups and VAT were generally the major contributing factors to this cost (Fig. 2).

In contrast, for the low-price drug category (anti-epileptics), the highest mark-up plus tax cost in terms of percentage of the ex-factory price was observed in Germany (187%), Norway (139%), Ireland (131%), Switzerland (123%), and Austria (112%). Fixed remuneration in terms of flat fees or fixed percentages were the key contributor to this mark-up cost in Germany (124% of the ex-factory price), while it was the pharmacy mark-up for both Ireland (80%) and Austria (80%), and consolidated distribution mark-up for Switzerland (118%). Interestingly, the impact of VAT (as % of the ex-factory price of the drug) accounted for approximately 50% for this drug category in Germany and Norway. Countries with the lowest mark-up plus tax cost included Croatia and Vietnam, where the mark-ups along with taxes accounted for 16% and 15% of the ex-factory price, respectively (Fig. 2). Absolute mark-ups for the three price categories are presented in Appendix Figures 1, 2, and 3.

**Fig. 3:**
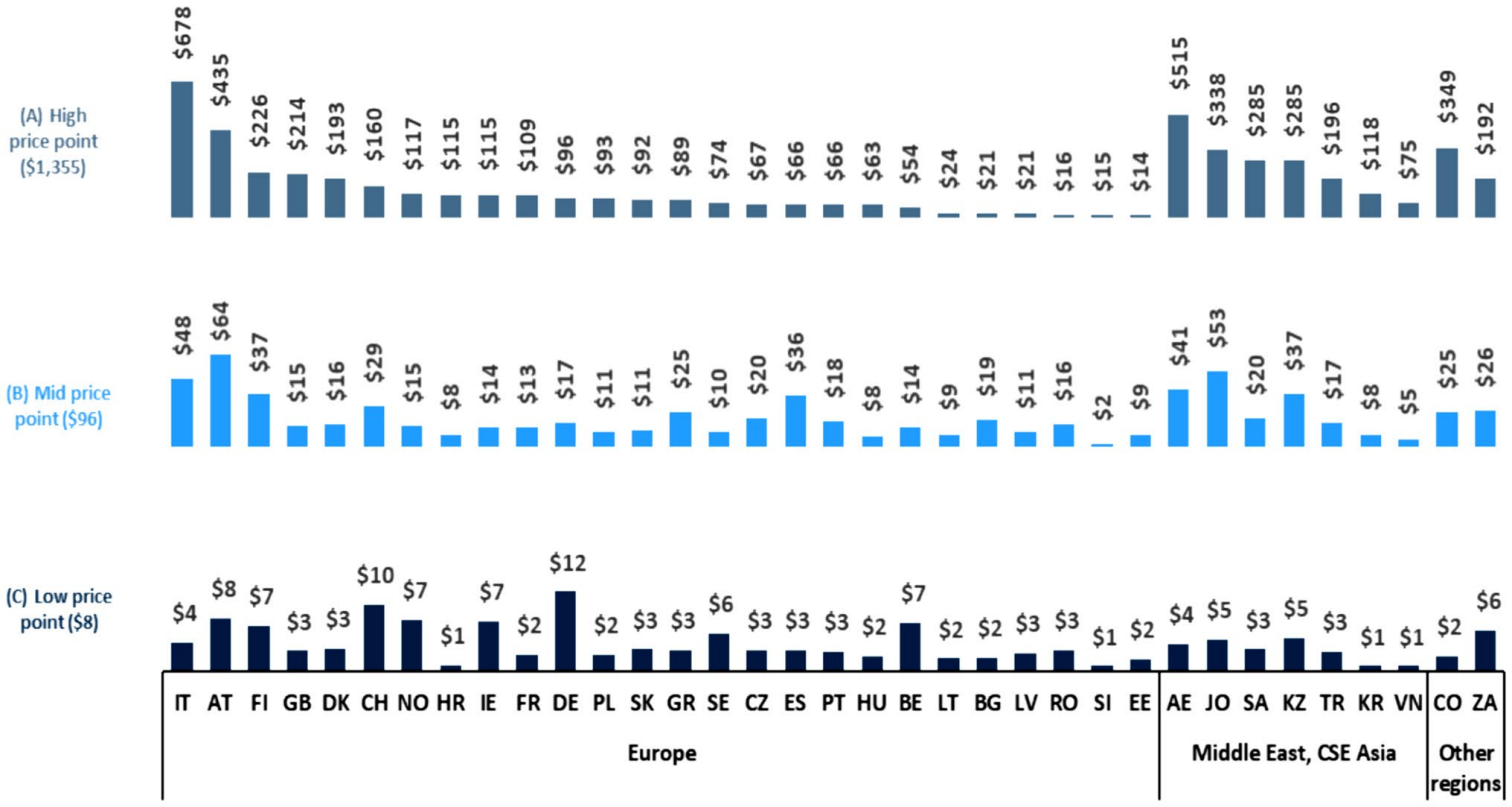
Overall absolute mark-ups (US$) excluding VAT at the high (US$1355), mid (US$96), and low (US$8) price points. Abbreviations: *AE* United Arab Emirates; *AT* Austria; *BE* Belgium; *BG* Bulgaria; *CH* Switzerland; *CO* Colombia; *CZ* Czech Republic; *DE* Germany; *DK* Denmark; *EE* Estonia; *ES* Spain; *FI* Finland; *FR* France; *GB* United Kingdom; *GR* Greece; *HR* Croatia; *HU* Hungary; *IE* Ireland; *IT* Italy; *JO* Jordan; *KR* Korea; *KZ* Kazakhstan; *LT* Lithuania; *LV* Latvia; *NO* Norway; *PL* Poland; *PT* Portugal; *RO* Romania; *SA* Saudi Arabia; *SE* Sweden; *SI* Slovenia; *SK* Slovakia; *TR* Turkey; *VN* Vietnam; *ZA* South Africa. Source. The authors based on IQVIA MIDAS^®^ data

The absolute values (US$) for mark-ups excluding VAT for the three drug price categories (Fig. 3) indicate large variation across the sample countries. For example, for monoclonal antibodies (ex-factory price: US$1355), mark-ups ranged from US$14 in Estonia to US$678 in Italy; the mark-ups were more than US$200 in 9 of the 35 countries (Italy, UAE, Austria, Colombia, Jordan, Saudi Arabia, Kazakhstan, Finland, and the UK) (Fig. 3).

**Fig. 4:**
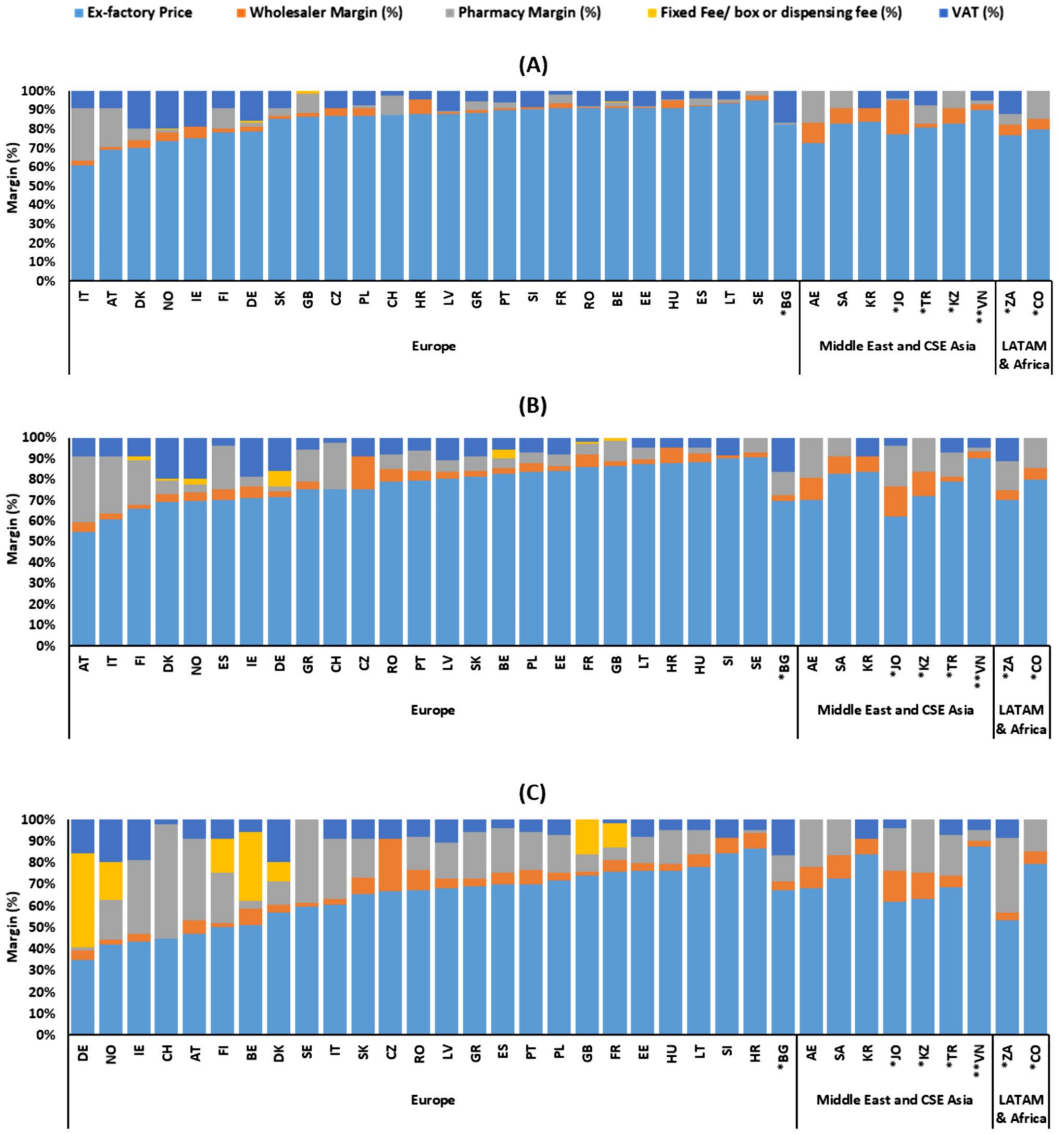
Comparison of overall margins (%) including VAT across the high (US$1355), mid (US$96), and low (US$8) price points. Abbreviations: *AE* United Arab Emirates; *AT* Austria; *BE* Belgium; *BG* Bulgaria; *CH* Switzerland; *CO* Colombia; *CSE* Central, South, and East; *CZ* Czech Republic; *DE* Germany; *DK* Denmark; *EE* Estonia; *ES* Spain; *FI* Finland; *FR* France; *GB* United Kingdom; *GR* Greece; *HR* Croatia; *HU* Hungary; *IE* Ireland; *IT* Italy; *JO* Jordan; *KR* Korea; *KZ* Kazakhstan; *LT* Lithuania; *LV* Latvia; *NO* Norway; *PL* Poland; *PT* Portugal; *RO* Romania; *SA* Saudi Arabia; *SE* Sweden; *SI* Slovenia; *SK* Slovakia; *TR* Turkey; *VN* Vietnam; *ZA* South Africa. *Upper middle-income countries; **Lower middle-income countries. All other markets are high income countries according to the World Bank classification [75]; For CZ and SI, a combined mark-up information was present for wholesale and pharmacy. In the figure, total mark-up is illustrated as wholesale mark-up. Source. The authors based on IQVIA MIDAS^®^ data

Figure 4 presents a comparative overview of overall margins (instead of mark-ups) including VAT across the three price categories indicating a general trend that the overall margins were highest at the lowest price point and vice-versa. The proportion of margins plus tax ranged from 39.4% (Italy) to 5.2% (Sweden) for the high-price category, from 46% (Austria) to 10% (Sweden) for the medium-price category, and from 65% (Germany) to 13% (Vietnam) in the low-price drug category. In three countries (Italy [39%], Colombia [20%], and Korea [16%]), the proportion of margins including VAT to the overall retail cost of the drug remained consistent irrespective of the ex-factory price level of the drug (Fig. 4). This can be explained by the fixed remuneration structure of the distribution chain that applies across the price range of different pharmaceutical products.

Sensitivity analysis showed similar trends as the primary analysis (Appendix Figures 4–10). Distribution mark-ups and taxes varied across the remaining price categories across different countries. The degree of variation was not different to the one that has already been reported in the preceding analysis. Additionally, due to capping on wholesale or pharmacy mark-ups, which is usually taking the form of a fixed remuneration by means of a flat fee, the absolute wholesale or pharmacy mark-ups did not change beyond a specific price point upon further increases in ex-factory prices (Austria, Bulgaria, Estonia, France, Germany, Hungary, Jordan, Lithuania, Romania, Slovenia, Spain, Sweden, and Switzerland).

## Discussion and policy implications

Assuming the same average price across settings for specific product archetypes, the results from this study suggest that retail prices for prescription drugs vary considerably across countries due to observed wide differences in distribution mark-ups and taxes. Significant variations in regulated wholesaler and pharmacy mark-ups, dispensing fees, and VAT rates were observed across different geographies.

First, we have identified three remuneration types in the distribution chain, one of which is “hybrid” as it combines elements from the other two types. The first is the regressive mark-up structure (price dependent), taking the form of regressive percentages, or regressive fees, or a combination of regressive percentages and fees, whereby both percentages and fees are used in declining order to remunerate the distribution chain (pursued by 11 countries in wholesale and 15 countries in retail distribution). The second type is fixed remuneration (non-price dependent) either on a percentage basis or on a fee basis (pursued by 19 countries in wholesale and 10 countries in retail distribution). The third (hybrid) remuneration type takes the form of a regressive mark-up with capping of prices and a single fixed fee beyond the cap (pursued by 5 countries in wholesale and 9 countries in retail distribution), i.e. price dependent till a specific price threshold and non-price dependent thereafter. Fixed remuneration seems to dominate in wholesaling (54%) while regressive mark-up structures dominate (43%) in retailing. From a policy standpoint, there is no preferred or optimal remuneration structure, although most countries in our sample apply price dependent remuneration structures, particularly at pharmacy level. The selection of remuneration structure and the size of remuneration seem to be a function of historical development coupled with the size of the distribution sector, particularly retail, in terms of outlets per 1000 population. Time series would be necessary to reveal how historically set remuneration may have changed in light of developments in drug distribution and the changing pattern of available drugs on the market. A further issue is that of incentivisation of professionals at pharmacy level. Cost-effective dispensing is prima facie incentivised through regressive mark up structures, but advice provided by pharmacists to patients may not be reflected in such structures. In a likely development where pharmacists take a more active role in patient advisory services, non-price dependent remuneration, such as fixed percentages or fixed fees may become more relevant or could be incorporated in some way into existing structures.

Second, the analysis of price components revealed that mark-ups applied to ex-factory prices varied significantly across countries for the three price categories. Mark-up regressivity has meant that mark-ups generally increased with a decrease in ex-factory prices. Mark-up plus tax as a proportion of ex-factory prices ranged from 5% (Sweden) to 65% (Italy) for the high-price category, 11% (Sweden, Slovenia, and Vietnam) to 84% (Austria) for the medium-price category, and 15% (Vietnam) to 187% (Germany) for the low-price category, highlighting the substantial additional cost beyond ex-factory prices to health systems and patients. Austria (pharmacy mark-up accounting for 46%, 59%, and 80% of the ex-factory price for the high, medium, and low price category, respectively) and Norway (VAT accounting for 27%, 29%, and 48% of the ex-factory price for the high, medium, and low price category, respectively) were consistently in the leading five countries in terms of mark-up and tax costs across the three price point categories. In general, pharmacy regressive mark-ups were the key contributor to the overall mark-up costs for the high-priced drugs whereas fixed remuneration along with VAT contributed significantly in the low-priced drug category. Overall, no specific trend was observed across the study countries in different income categories or regions in terms of regulations implemented for mark-ups. Our calculations highlight the moderate-to-significant cost of distribution and taxation to health system budgets beyond ex-factory prices of drugs.

Third, average margins and taxes ranged from 5% (Sweden, for high-priced products) to 65% (Germany, for low-priced products). Typically, the impact of margins and taxes decreases in relative terms as the cost of prescription drugs increases; the highest impact is shown by the cheapest products across the board, with few exceptions, where the distribution and taxation cost is the same across product price bands (Italy, Colombia, Korea) (Appendix Table 3).

Fourth, countries are resorting to fairly complex regressive structures for both wholesale and pharmacy mark-ups with multiple bands. One notable trend is the number of countries pursuing fixed remuneration, especially flat fee (*n* = 9), particularly in the retail sector and for both expensive and very low-cost drugs. The former would attract a very high fee if a percentage remuneration had been adopted, while the latter constitute the bulk of volume of drugs dispensed in most, if not all, countries. While markups and fixed remuneration exist to guarantee minimum and fair income levels to the distribution chain, this practice elevates the cost of distribution to the health care system considerably. Additionally, the variability observed across countries suggests that other factors are at play influencing the level of distribution remuneration. The density of distribution (particularly retail) outlets may be determined by regulatory policies related to geographic or population criteria. Equally, prevailing policies on horizontal or vertical integration, shape market structure and levels of concentration, and may, in turn, influence overall remuneration levels and may have a bearing on what might otherwise constitute an efficient market structure. Earlier evidence suggests that the higher the number of outlets on the market, the greater the need for higher mark ups in order to increase the viability of the retail sector [7, 8].

Fifth, the WHO guidelines on country pharmaceutical pricing policies published in 2020 provide recommendations across a variety of pricing policies including distribution mark-up regulations, and tax exemption or tax reduction for pharmaceutical products. The guidelines suggest that the mark-ups at various levels of the pharmaceutical distribution chain should be regressive in nature and countries should ensure a transparent system for prices and methods while setting up mark-up structures [11]. The body of evidence from this study largely conforms with this recommendation in that a large number of countries pursue regressive markups. However, fixed remuneration (either a flat fee or a fixed percentage) was also observed in 19 countries at wholesale level and in 10 countries at retail level, indicating a need to place a cap on overall distribution costs. This type of practice is likely to become increasingly more relevant in the future as the number of high cost and highly specialised products and personalised medicines proliferate. Such products may require different arrangements and the traditional distribution model may prove inadequate to serve the needs of patients, health systems, and the distribution chain. Moving towards more sophisticated models of distribution may prove unavoidable. Alternative wholesaling models, either reduced wholesaling (relying on preferential contracting of a limited number of wholesalers to distribute highly specialised products) may offer an alternative to the “competitive” model, where all wholesalers have the right to distribute pharmaceutical products. Alternatively, agency or direct-to-pharmacy models, may offer advantages to manufacturers in that the ownership of the stock remains with them until it reaches its destination (e.g., a retail outlet or the patient’s home).

Sixth, while the vast majority of the study countries (83%) apply VAT, 63% recognise that prescription drugs should be treated differently and, therefore, apply a lower VAT rate than the standard for other commodities. Taxes on prescription drugs constitute stealth taxes in that they reduce available resources from health budgets, which could otherwise be expended on providing meaningful care to patients. A wide variability in taxation across the countries and differences in VAT rates for prescription drugs vs. other commodities was observed in this study, suggesting that optimal taxation systems should be designed which may help reduce the prices of prescription drugs and therefore burden on payers and healthcare systems. The WHO suggests that countries consider exempting essential drugs and active pharmaceutical ingredients from taxation, with measures to ensure their implementation results in lower drug prices [11]. Considering the high volume and low cost of these drugs, it may be a reasonable way forward for this to be implemented, offering some relief to health budgets.

Seventh, the evidence presented captures items that are visible and amenable to analysis, nevertheless, it does not capture a number of issues which impact rents within the distribution chain: (a) there exist discounting practices between wholesalers and retailers, or manufacturers and wholesalers, or manufacturers and retailers, which can be significant [7, 8, 82, 83]. These often take the form of price discounts (where allowed), but there is extensive volume discounting as well. These constitute additional forms of income and often lie beyond the control of governmental organisations, but can be addressed as evidence suggests. In the UK, for example, the clawback, is a mechanism whereby the government reimburses pharmacies for the cost of pharmacy-dispensed prescription drugs, but retains approximately 10% of the reimbursement cost, because of the discounting that goes on between manufacturers, wholesalers and pharmacies. In other settings, tenders in the outpatient (pharmacy) setting, force manufacturers to offer their lowest price to health insurers, thus eliminating the scope of discounting to the distribution chain. Equally, there may be parallel trade (under specific circumstances), which also affects the way the market operates and could create conditions of shortage in exporting countries; the latter are a threat to public health and can be addressed through export bans and enforcing such measures that the distribution chain should first and foremost satisfy the need of the local market prior to exporting products; (b) it is not clear, particularly for expensive products, what happens to distribution mark-ups if a risk sharing agreement enters into force that results in a difference between list and net price and how distribution chain income is affected; similarly, where procurement via tendering takes place, the distribution mark up (wholesale and pharmacy) may be adjusted downwards to the tender price (as was the case originally in the Netherlands through the introduction of preference-based policies [84]), or remain the same based on the market price (e.g. Germany [84]); if anything, distribution chain remuneration should be based on net prices. Nevertheless, this presents a number of implementation challenges, such as compromising the confidentiality of managed entry agreements (MEAs), facilitating formal or informal “net reference pricing” and placing a burden on manufacturers to cover the overcharged portion by retail distributors, i.e. the mark-up on the difference between list and net price; all these pose a threat on the conclusion of a MEA and the concomitant access to patients; and (c) distribution chain remuneration (and its size) is also dependent on the nature of regulation (set up restrictions, presence of geographical or population criteria, ownership criteria, etc.), the nature of competition in pharmaceutical markets (concentration ratio), the ability of the distribution chain to consolidate horizontally or vertically, and the different market dynamics between originator in-patent medicines, where no generic is available, and the off-patent segment, where originator brands and generics co-exist, all of which are factors that deserve to be factored in when designing distribution chain remuneration models.

Finally, very few previous studies have demonstrated a positive impact of regulating distribution mark-ups on reducing pharmaceutical expenditures. In Ireland, one study evaluated the impact of various cost-containment interventions on the CDS between 2005 and 2010. The results showed statistically significant reductions in expenditure for patented and generic products through mark-up regulations (*p* < 0.05) [85]. Similarly, in China, a study concluded that a Zero Mark-up Drug (ZMD) policy was effective in regulating drug-related expenditures and achieved better intervention effects than the Fixed Percent Mark-up Drug policy (FPM) [86]. However, the regulation of mark-ups may have unintended consequences, i.e., impact on the viability of wholesaler and retailer densities, particularly in remote areas, due to lower incentivisation, unless other incentives are in place for this purpose, for example, through the dispensing doctors particularly in remote areas (e.g., UK, Switzerland), or schemes providing funding to support pharmacies to stay open in rural or remote areas in order to provide accessible primary care. Therefore, governments and policy-makers must carefully calibrate distribution mark-ups based on local need, ensure implementation of these regulations, and frequently monitor their impact on prices of drugs as well as on the overall healthcare expenditure.

The gradual shift towards niche products, the declining cost of generic drugs through tendering, and the trend towards personalised medicine will affect the profitability of existing distribution structures and contribute to further changes in the market structure of the distribution chain. Horizontal integration amongst wholesalers or retailers has already taken place, particularly in the former, whereas in the latter it is subject to national legislation relating to the establishment of retail chains [7]. Similarly, vertical integration, where wholesalers take over retailers or vice versa, has expanded in recent years, but may be constrained based on national legislation [7, 8]. At the same time, new models of wholesale distribution have started to appear on the market, such as direct-to-pharmacy (DTP) (agency model), where the distributor is a logistics provider, or the reduced wholesaler model (RWM), whereby manufacturers contract with a small number of wholesalers to distribute all or part of their product portfolio. Uptake of these models looks variable both at country level, where the principle of public service obligation does not apply, but also at company level, with some companies subscribing to the agency model (e.g., Pfizer in 2007 [87] and AstraZeneca in 2019, both in the UK) despite concerns being raised about the resilience of the pharmaceutical supply chain [88], while GSK announced in 2018 [89] it would be moving away from the agency model and follow the reduced wholesaler model (specifically three wholesalers, also in the UK). Changes in the structure and operation of the distribution chain are likely to influence competition and, unavoidably, will impact remuneration. Broader issues, such as these relating to service levels of the distribution chain and, potentially, shortages, may also arise.

### Limitations and future research

Our analysis is not without limitations. First, it does not capture the in-patient market, which altogether has different characteristics in that pharmacy mark-ups and taxes may not apply. Moreover, the study is focused on evaluating traditional distribution channels (wholesale to retail/community pharmacy) and does not consider any emerging practices including the direct-to-public model, the direct-to-hospital model, or wholesaler and pharmacy managed by the same organization. Second, we have reflected on retail community pharmacy mark-ups in our analysis. In some countries (e.g., Switzerland and Austria), there may be other dispensing actors (i.e., dispensing doctors) involved, however, their remuneration (if any) was not considered in this study. Third, our study focuses on data collected from countries that are known to regulate the distribution channel and/or taxes and does not include countries that do not regulate the distribution of prescription drugs. The latter are known to have higher distribution mark-ups and if taxes are also imposed, their absolute impact on health systems and/or patients can be higher [16]. Assessing the impact of distribution mark ups in unregulated settings could be the subject of a future study. Although we have strived to include as many countries in our analysis as possible, based on the scope, it is likely that some countries with regulated distribution remuneration structures are not included, since country inclusion in the study was based on the availability of information regarding distribution and taxation. Fourth, the source documents (and the information therein) may relate to different time periods and may have been published earlier than our data collection period, however, they represent the latest information available on the respective sources across the selected countries. Fifth, we prioritized distribution remuneration information for prescription drugs which are reimbursed, distributed through retail channel/pharmacy, are patented, and are available as non-hospital drugs, among others. For example, regulations may be different when prescription drugs are not reimbursed (but are available) or when the drugs in question are available over the counter (OTC). Mark-ups in the former case are impacting patients directly, if the products are available despite reimbursement not being granted, while in the second case, mark-ups may be unregulated and, may even be higher as a result. Sixth, we selected weighted average ex-factory prices as price proxies of different price categories. The selection of the initial product classes and their classification as “high-”, “medium-” or “low-price” classes is based on empirical observation of prices associated with the products in these categories. Although this was done in a systematic way, other therapeutic classes could have been selected to represent the price points required. In order to account for likely variations in our results, sensitivity analysis was conducted. Seventh, we assumed the same ex-factory price levels for all study countries, although prices would differ based on real-world practice. However, this was undertaken to understand the impact of mark-ups and taxes and exclude the impact of any other potential factors on retail prices. Eighth, the actual final prices may be relatively lower vs. those estimated using distribution remuneration and taxes due to discounting and price negotiations, which we are not able to capture. Additionally, in our study, the data shown do not capture discounting practices that may exist between manufacturers, wholesalers and retailers; these are allowed in many—if not most—settings, can be price- or volume-related and constitute a source of income for certain parts of the distribution chain (particularly for retailers). In some countries (e.g., Spain or Italy), there are statutory pharmacy discounts regulated by law, however, such discounts are not considered in this study. Additionally, various other access schemes i.e., MEAs are not included in the scope of the study. Ninth, while it might be desirable to derive “average” wholesale and/or pharmacy mark-ups from this analysis, this is not possible considering the complex structure of distribution policies, as in the vast majority of cases, wholesale and pharmacy mark-ups are regressive and often fused with flat fees or fixed percentage remuneration and are heavily dependent on the price levels of individual drugs. Finally, in some cases the mark-ups are not clear as there are arrangements regarding remuneration of the distribution chain, which rely on contractual agreements between national competent authorities and distribution chain representatives (e.g., the UK); in this case, it is not clear or visible what the relevant distribution mark-ups are.

The evidence and its complexity provided in this study and the associated limitations, highlight a number of areas to undertake further research in the future. These areas include and are by no means limited to: first, how different models of wholesale distribution, such as DTP or reduced wholesaler models, perform, where they are allowed; second, understanding from a health care system and, possibly also from a geographical perspective, what drives differences in margins across settings; third, assess the impact of policies relating to clawback and tendering in the context of outpatient drugs and what effect the latter practice may have on pharmacy remuneration; fourth, study the market dynamics and any differences that may exist, between originator in-patent medicines, with no generic competitor, and the off-patent segment, where off-patent originators and generics co-exist; fifth, the degree of horizontal and vertical integration among interested parties (manufacturers, wholesalers and pharmacies) and how it affects remuneration but also broader aspects of competition in the distribution system; and, finally, whether the health care financing model has any effect on the type of distribution remuneration across settings.

## Conclusion

We have conducted a review and analysis of distribution chain remuneration and taxation systems for prescription drugs with focus on the retail sector. Having identified three remuneration types of the wholesale and retail distribution chain across 35 countries, we find that all three are represented in country preferences. The general trend remains to remunerate wholesale and retail distribution on a regressive mark-up basis, although the presence of fixed fees and fixed percentages is also very high. The observed cost of distribution mark-ups and taxes can result in very high retail prices for drugs, depending on product price range, and this cost is inversely related to the ex-factory prices of individual drugs. Overall, margins also vary significantly by country and product price range ranging from as low as 5% of the retail price for high price products to as high as 65% of the retail price for low-price products. Although the level of remuneration of the distribution chain raises efficiency and overall affordability questions at health system level, these need to be viewed in conjunction with the regulatory framework shaping market structure, including population, geographic and ownership criteria, as well as policies and practices on horizontal and vertical integration. One area where the overall cost of prescription drugs could be reduced with immediate effect is taxation. Some countries have applied zero or low VAT on prescription drugs, while others still apply standard rates. Zero VAT on prescription drugs could go some way to alleviating immediate fiscal pressures on health budgets, whilst avoiding resource re-allocation from health to other sectors. Further research is warranted to broaden the scope of this analysis by including more geographies and comparison of distribution and taxation systems.

## Supplementary Information

Below is the link to the electronic supplementary material.

Supplementary file1 (DOCX 260 KB)
