# Supplementary material for: An assessment of the implications of distribution remuneration and taxation policies on the final prices of prescription medicines: evidence from 35 countries
Source: Eur J Health Econ. 2024 Sep 19;26(3):513–36. doi: 10.1007/s10198-024-01706-x (PMC11937219; doi:10.1007/s10198-024-01706-x)
Supplement: Supplementary file 1 — Supplementary file1 (DOCX 260 KB) [file 10198_2024_1706_MOESM1_ESM.docx]

# Appendix

Appendix Table 1: Pharmaceutical market value (ex-factory level) by ATC 1 category, 2020

| **ATC1 class** | **2020 sales (in billion US$)** | **Percentage of total sales (%)** |
| --- | --- | --- |
| **Overall ATC1 classes** | **1,141.00** | **100%** |
| L (Antineoplastic and immunomodulating agents) | 260.87 | 23% |
| A (Alimentary tract and metabolism) | 177.02 | 16% |
| N (Nervous system) | 146.55 | 13% |
| J (General anti-infectives systemic) | 116.65 | 10% |
| C (Cardiovascular system) | 77.62 | 7% |
| R (Respiratory system) | 75.79 | 7% |
| B (Blood and blood forming organs) | 75.56 | 7% |
| M (Musculo-skeletal system) | 39.44 | 3% |
| G (Genito-urinary system and sex hormones) | 33.34 | 3% |
| S (Sensory organs) | 29.74 | 3% |
| V (Various) | 26.41 | 2% |
| D (Dermatologicals) | 26.72 | 2% |
| H (Systemic hormonal preparations) | 23.17 | 2% |
| T (Diagnostic agents) | 16.00 | 1% |
| K (Hospital solutions) | 14.25 | 1% |
| P (Parasitology) | 1.88 | 0% |

**Source**: IQVIA MIDAS® data.

Appendix Table 2: Weighted ex-factory prices for leading ten product classes based on sales

| **ATC 3 class** | **Global sales (Billion US$)** | **Contribution to total sales (%)** | **Ex-factory price in select countries*** | | | **Category for comparison** |
| --- | --- | --- | --- | --- | --- | --- |
|  |  |  | **Minimum** | **Maximum** | **Weighted average** |  |
| L1G (Monoclonal antibody antineoplastics) | 67 | 6% | $262 | $2,529 | $1,355 | **High priced-drugs (price category 1)** |
| L4B (Anti-TNF products) | 55 | 5% | $140 | $1,928 | $566 |  |
| L1H (Protein kinase inhibitor antineoplastics) | 40 | 4% | $48 | $3,648 | $191 |  |
| A10S (GLP-1 agonist anti-diabetics) | 22 | 2% | $20 | $233 | $96 | **Medium priced-drugs (category 2)** |
| B1F (Direct factor XA inhibitors) | 30 | 3% | $14 | $164 | $43 |  |
| A10C (Human insulins and analogs) | 41 | 4% | $8 | $79 | $36 |  |
| N3A (Anti-epileptics) | 17 | 2% | $4 | $39 | $8 | **Low priced-drugs (category 3)** |
| C10A (cholesterol & triglycerides regulating preparations) | 13 | 1% | $2 | $55 | $4 |  |
| A2B (Antiulcerants) | 16 | 1% | $1 | $25 | $3 |  |
| N2B (Non-narcotics and antipyretics) | 11 | 1% | $0 | $5 | $2 |  |

**Note**: *Ex-factory price in selected 35 countries for analysis.

**Source**: The authors based on IQVIA MIDAS^®^ data.

Appendix Table 3 – Average margins (including VAT) for the three price categories across 35 countries

| **Country/product type** | **High price products** | **Medium-price products** | **Low-price products** |
| --- | --- | --- | --- |
| IT | 39% | 39% | 39% |
| AT | 31% | 46% | 53% |
| DK | 30% | 31% | 43% |
| NO | 26% | 31% | 58% |
| IE | 25% | 29% | 57% |
| FI | 22% | 34% | 50% |
| DE | 22% | 29% | 65% |
| SK | 15% | 19% | 34% |
| GB | 14% | 14% | 26% |
| CZ | 13% | 25% | 33% |
| PL | 13% | 17% | 28% |
| CH | 13% | 25% | 55% |
| HR | 12% | 12% | 14% |
| LV | 12% | 20% | 32% |
| GR | 11% | 25% | 31% |
| PT | 10% | 21% | 30% |
| SI | 10% | 10% | 16% |
| FR | 9% | 14% | 24% |
| RO | 9% | 21% | 33% |
| BE | 9% | 18% | 49% |
| EE | 9% | 16% | 24% |
| HU | 9% | 12% | 24% |
| ES | 8% | 30% | 30% |
| LT | 6% | 13% | 22% |
| SE | 5% | 10% | 40% |
| BG | 18% | 31% | 33% |
| AE | 28% | 30% | 32% |
| SA | 17% | 17% | 28% |
| KR | 16% | 16% | 16% |
| JO | 23% | 38% | 38% |
| TR | 19% | 21% | 31% |
| KZ | 17% | 28% | 37% |
| VN | 10% | 10% | 13% |
| ZA | 23% | 30% | 47% |
| CO | 20% | 20% | 20% |

**Abbreviations**: AE: United Arab Emirates; AT: Austria; BE: Belgium; BG: Bulgaria; CSE: Central, South, and East Asia; CH: Switzerland; CO: Colombia; CZ: Czech Republic; DE: Germany; DK: Denmark; EE: Estonia; ES: Spain; FI: Finland; FR: France; GB: United Kingdom; GR: Greece; HR: Croatia; HU: Hungary; IE: Ireland; IT: Italy; JO: Jordan; KR: Korea; KZ: Kazakhstan; LATAM: Latin America; LT: Lithuania; LV: Latvia; NO: Norway; PL: Poland; PT: Portugal; RO: Romania; SA: Saudi Arabia; SE: Sweden; SI: Slovenia; SK: Slovakia; TR: Turkey; VN: Vietnam; ZA: South Africa.

**Source**: The authors based on IQVIA MIDAS^®^ data.

Appendix Figure 1: Impact of mark-ups on price of monoclonal antibodies (assuming a fixed ex-factory price at US$1,355)^1^

**Abbreviations**: AE: United Arab Emirates; AT: Austria; BE: Belgium; BG: Bulgaria; CH: Switzerland; CO: Colombia; CZ: Czech Republic; DE: Germany; DK: Denmark; EE: Estonia; ES: Spain; FI: Finland; FR: France; GB: United Kingdom; GR: Greece; HR: Croatia; HU: Hungary; IE: Ireland; IT: Italy; JO: Jordan; KR: Korea; KZ: Kazakhstan; LT: Lithuania; LV: Latvia; NO: Norway; PL: Poland; PT: Portugal; RO: Romania; SA: Saudi Arabia; SE: Sweden; SI: Slovenia; SK: Slovakia; TR: Turkey; VN: Vietnam; ZA: South Africa.

**Notes**: *Upper middle-income countries;

**Lower middle-income countries. All other markets are high income countries according to the World Bank classification (80); For CZ and SI, a combined mark-up information was present for wholesale and pharmacy. In the figure, total mark-up is illustrated as wholesale mark-up.

^1^ Values on top of each column represent final retail prices.

Appendix Figure 2: Impact of mark-ups on price of GLP-1 agonists (assuming a fixed ex-factory price at US$96)^1^

**Abbreviations**: AE: United Arab Emirates; AT: Austria; BE: Belgium; BG: Bulgaria; CH: Switzerland; CO: Colombia; CZ: Czech Republic; DE: Germany; DK: Denmark; EE: Estonia; ES: Spain; FI: Finland; FR: France; GB: United Kingdom; GR: Greece; HR: Croatia; HU: Hungary; IE: Ireland; IT: Italy; JO: Jordan; KR: Korea; KZ: Kazakhstan; LT: Lithuania; LV: Latvia; NO: Norway; PL: Poland; PT: Portugal; RO: Romania; SA: Saudi Arabia; SE: Sweden; SI: Slovenia; SK: Slovakia; TR: Turkey; VN: Vietnam; ZA: South Africa.

**Notes**: *Upper middle-income countries;

**Lower middle-income countries. All other markets are high income countries according to the World Bank classification (80); For CZ and SI, a combined mark-up information was present for wholesale and pharmacy. In the figure, total mark-up is illustrated as wholesale mark-up.

^1^ Values on top of each column represent final retail prices.

Appendix Figure 3: Impact of mark-ups on price of anti-epileptics (assuming a fixed ex-factory price at US$8)^1^

**Abbreviations**: AE: United Arab Emirates; AT: Austria; BE: Belgium; BG: Bulgaria; CH: Switzerland; CO: Colombia; CZ: Czech Republic; DE: Germany; DK: Denmark; EE: Estonia; ES: Spain; FI: Finland; FR: France; GB: United Kingdom; GR: Greece; HR: Croatia; HU: Hungary; IE: Ireland; IT: Italy; JO: Jordan; KR: Korea; KZ: Kazakhstan; LT: Lithuania; LV: Latvia; NO: Norway; PL: Poland; PT: Portugal; RO: Romania; SA: Saudi Arabia; SE: Sweden; SI: Slovenia; TR: Turkey; VN: Vietnam; ZA: South Africa.

**Notes**: *Upper middle-income countries;

**Lower middle-income countries. All other markets are high income countries according to the World Bank classification (80); For CZ and SI, a combined mark-up information was present for wholesale and pharmacy. In the figure, total mark-up is illustrated as wholesale mark-up.

^1^ Values on top of each column represent final retail prices.

Appendix Figure 4: Impact of mark-ups on price of A10C (Human insulin + analogues) (assuming a fixed ex-factory price at US$36.44)^1^

**Abbreviations**: AE: United Arab Emirates; AT: Austria; BE: Belgium; BG: Bulgaria; CH: Switzerland; CO: Colombia; CZ: Czech Republic; DE: Germany; DK: Denmark; EE: Estonia; ES: Spain; FI: Finland; FR: France; GB: United Kingdom; GR: Greece; HR: Croatia; HU: Hungary; IE: Ireland; IT: Italy; JO: Jordan; KR: Korea; KZ: Kazakhstan; LT: Lithuania; LV: Latvia; NO: Norway; PL: Poland; PT: Portugal; RO: Romania; SA: Saudi Arabia; SE: Sweden; SI: Slovenia; TR: Turkey; VN: Vietnam; ZA: South Africa.

**Notes**: *Upper middle-income countries;

**Lower middle-income countries. All other markets are high income countries according to the World Bank classification (80); For CZ and SI, a combined mark-up information was present for wholesale and pharmacy. In the figure, total mark-up is illustrated as wholesale mark-up.

^1^ Values on top of each column represent final retail prices.

Appendix Figure 5: Impact of mark-ups on price of A2B (Anti-ulcerants) (assuming a fixed ex-factory price at US$3.5)^1^

**Abbreviations**: AE: United Arab Emirates; AT: Austria; BE: Belgium; BG: Bulgaria; CH: Switzerland; CO: Colombia; CZ: Czech Republic; DE: Germany; DK: Denmark; EE: Estonia; ES: Spain; FI: Finland; FR: France; GB: United Kingdom; GR: Greece; HR: Croatia; HU: Hungary; IE: Ireland; IT: Italy; JO: Jordan; KR: Korea; KZ: Kazakhstan; LT: Lithuania; LV: Latvia; NO: Norway; PL: Poland; PT: Portugal; RO: Romania; SA: Saudi Arabia; SE: Sweden; SI: Slovenia; TR: Turkey; VN: Vietnam; ZA: South Africa.

**Notes**: *Upper middle-income countries;

**Lower middle-income countries. All other markets are high income countries according to the World Bank classification (80); For CZ and SI, a combined mark-up information was present for wholesale and pharmacy. In the figure, total mark-up is illustrated as wholesale mark-up.

^1^ Values on top of each column represent final retail prices.

Appendix Figure 6: Impact of mark-ups on price of B1F (Direct factor XA inhibitors) (assuming a fixed ex-factory price at US$43.26)^1^

**Abbreviations**: AE: United Arab Emirates; AT: Austria; BE: Belgium; BG: Bulgaria; CH: Switzerland; CO: Colombia; CZ: Czech Republic; DE: Germany; DK: Denmark; EE: Estonia; ES: Spain; FI: Finland; FR: France; GB: United Kingdom; GR: Greece; HR: Croatia; HU: Hungary; IE: Ireland; IT: Italy; JO: Jordan; KR: Korea; KZ: Kazakhstan; LT: Lithuania; LV: Latvia; NO: Norway; PL: Poland; PT: Portugal; RO: Romania; SA: Saudi Arabia; SE: Sweden; SI: Slovenia; TR: Turkey; VN: Vietnam; ZA: South Africa.

**Notes**: *Upper middle-income countries;

**Lower middle-income countries. All other markets are high income countries according to the World Bank classification (80); For CZ and SI, a combined mark-up information was present for wholesale and pharmacy. In the figure, total mark-up is illustrated as wholesale mark-up.

^1^ Values on top of each column represent final retail prices.

Appendix Figure 7: Impact of mark-ups on price of C10A (Cholesterol & Triglyceride regulator) (assuming a fixed ex-factory price at US$4.63)^1^

**Abbreviations**: AE: United Arab Emirates; AT: Austria; BE: Belgium; BG: Bulgaria; CH: Switzerland; CO: Colombia; CZ: Czech Republic; DE: Germany; DK: Denmark; EE: Estonia; ES: Spain; FI: Finland; FR: France; GB: United Kingdom; GR: Greece; HR: Croatia; HU: Hungary; IE: Ireland; IT: Italy; JO: Jordan; KR: Korea; KZ: Kazakhstan; LT: Lithuania; LV: Latvia; NO: Norway; PL: Poland; PT: Portugal; RO: Romania; SA: Saudi Arabia; SE: Sweden; SI: Slovenia; TR: Turkey; VN: Vietnam; ZA: South Africa.

**Notes**: *Upper middle-income countries;

**Lower middle-income countries. All other markets are high income countries according to the World Bank classification (80); For CZ and SI, a combined mark-up information was present for wholesale and pharmacy. In the figure, total mark-up is illustrated as wholesale mark-up.

^1^ Values on top of each column represent final retail prices.

Appendix Figure 8: Impact of mark-ups on price of L1H (Protein kinase inhibitor ANT) (assuming a fixed ex-factory price at US$191.8)^1^

**Abbreviations**: AE: United Arab Emirates; AT: Austria; BE: Belgium; BG: Bulgaria; CH: Switzerland; CO: Colombia; CZ: Czech Republic; DE: Germany; DK: Denmark; EE: Estonia; ES: Spain; FI: Finland; FR: France; GB: United Kingdom; GR: Greece; HR: Croatia; HU: Hungary; IE: Ireland; IT: Italy; JO: Jordan; KR: Korea; KZ: Kazakhstan; LT: Lithuania; LV: Latvia; NO: Norway; PL: Poland; PT: Portugal; RO: Romania; SA: Saudi Arabia; SE: Sweden; SI: Slovenia; TR: Turkey; VN: Vietnam; ZA: South Africa.

**Notes**: *Upper middle-income countries;

**Lower middle-income countries. All other markets are high income countries according to the World Bank classification (80); For CZ and SI, a combined mark-up information was present for wholesale and pharmacy. In the figure, total mark-up is illustrated as wholesale mark-up.

^1^ Values on top of each column represent final retail prices.

Appendix Figure 9: Impact of mark-ups on price of L4B (Anti-TNF products) (assuming a fixed ex-factory price at US$566.37)^1^

**Abbreviations**: AE: United Arab Emirates; AT: Austria; BE: Belgium; BG: Bulgaria; CH: Switzerland; CO: Colombia; CZ: Czech Republic; DE: Germany; DK: Denmark; EE: Estonia; ES: Spain; FI: Finland; FR: France; GB: United Kingdom; GR: Greece; HR: Croatia; HU: Hungary; IE: Ireland; IT: Italy; JO: Jordan; KR: Korea; KZ: Kazakhstan; LT: Lithuania; LV: Latvia; NO: Norway; PL: Poland; PT: Portugal; RO: Romania; SA: Saudi Arabia; SE: Sweden; SI: Slovenia; TR: Turkey; VN: Vietnam; ZA: South Africa.

**Notes**: *Upper middle-income countries;

**Lower middle-income countries. All other markets are high income countries according to the World Bank classification (80); For CZ and SI, a combined mark-up information was present for wholesale and pharmacy. In the figure, total mark-up is illustrated as wholesale mark-up.

^1^ Values on top of each column represent final retail prices.

Appendix Figure 10: Impact of mark-ups on price of N2B (Non-narcotic analgesics) (assuming a fixed ex-factory price at US$1.66)^1^

**Abbreviations**: AE: United Arab Emirates; AT: Austria; BE: Belgium; BG: Bulgaria; CH: Switzerland; CO: Colombia; CZ: Czech Republic; DE: Germany; DK: Denmark; EE: Estonia; ES: Spain; FI: Finland; FR: France; GB: United Kingdom; GR: Greece; HR: Croatia; HU: Hungary; IE: Ireland; IT: Italy; JO: Jordan; KR: Korea; KZ: Kazakhstan; LT: Lithuania; LV: Latvia; NO: Norway; PL: Poland; PT: Portugal; RO: Romania; SA: Saudi Arabia; SE: Sweden; SI: Slovenia; TR: Turkey; VN: Vietnam; ZA: South Africa.

**Notes**: *Upper middle-income countries;

**Lower middle-income countries. All other markets are high income countries according to the World Bank classification (80); For CZ and SI, a combined mark-up information was present for wholesale and pharmacy. In the figure, total mark-up is illustrated as wholesale mark-up.

^1^ Values on top of each column represent final retail prices.
